# Portfoliobasiertes Lernen in der Chirurgie: Kompetenzen „sichtbar“ machen

**DOI:** 10.1007/s00104-022-01758-w

**Published:** 2022-11-23

**Authors:** S. Herbstreit, L. Hille, F. Rademacher, M. Burggraf, B. Mester, M. Dudda

**Affiliations:** Zentrum für Muskuloskelettale Chirurgie, Klinik für Unfall‑, Hand- und Wiederherstellungschirurgie am Universitätsklinikum Essen, Medizinische Fakultät Universität Duisburg-Essen, Hufelandstr. 55, 45147 Essen, Deutschland

**Keywords:** Blockpraktikum, Kompetenzorientierte Lehre, Chirurgie im Medizinstudium, Portfolio, Reflexion, Block practical training, Competence orientation, Undergraduate surgical education, Portfolio, Reflection

## Abstract

**Hintergrund:**

Durch eine zunehmende Kompetenzorientierung des Medizinstudiums und der Weiterbildung werden chirurgische Currikula vielerorts angepasst. Diese sollten, neben chirurgischem Wissen und praktischen Fertigkeiten, auch Kompetenzen zu Differenzialdiagnostik und -therapie vermitteln. Die Vermittlung chirurgischen Wissens durch Vorlesungen und Seminare und der Nachweis praktischer Fertigkeiten, z. B. mithilfe des Einsatzes von Logbüchern im Blockpraktikum (BP) Chirurgie, führt nur eingeschränkt zu einer aktiven Auseinandersetzung mit den chirurgischen Kompetenzen zu Differenzialdiagnostik und -therapie. Ein reflexionsbasiertes Portfolio kann, durch die eigenständige schriftliche Bearbeitung chirurgischer Themen eine aktive Auseinandersetzung mit den Kompetenzen ermöglichen und verspricht einen höheren Lerneffekt. Bei der Implementierung eines solchen Portfolios im Rahmen des Tätigkeitsnachweises im BP wurden die Effekte auf den Kompetenzerwerb und auf die Art und Weise des Lernens untersucht.

**Material und Methoden:**

Mit einer Kombination aus einer quantitativen und einer qualitativen Methode („mixed methods“) haben wir den Einsatz eines Logbuchs im BP Chirurgie mit dem Einsatz eines reflexionsbasierten Portfolios verglichen. Mittels Fragebogenerhebungen vor und nach dem BP erfolgte durch die Studierenden eine Selbsteinschätzung von Kompetenzen. Im Rahmen von Fokusgruppeninterviews anhand eines Leitfadens, mit Diskussionen unter Studierenden, haben wir die unterschiedlichen Wege des Kompetenzerwerbs untersucht. Zusätzlich wurden die Prüfungs- und Evaluationsergebnisse beider Kohorten verglichen.

**Ergebnisse und Diskussion:**

Der in der Selbsteinschätzung erhobene Kompetenzerwerb der Studierenden und die Prüfungs- und Evaluationsergebnisse zeigten im Vergleich beider Kohorten keinen Unterschied. Im Rahmen der Fokusgruppeninterviews konnten wir zeigen, dass in der Wahrnehmung der Studierenden chirurgische Kompetenzen mithilfe eines reflexionsbasierten Portfolio sichtbarer und damit eindeutiger gemacht werden können. Zusätzlich wurde selbstreguliertes Lernen der Studierenden gefördert, ohne dass praktische Fertigkeiten vernachlässigt wurden. Eine stärkere Supervision und Anleitung durch Mentor:innen wurde in beiden Gruppen gefordert.

## Abstract (en)

**Background:**

Due to an increasing competence orientation of medical studies, surgical curricula are being adapted in many places. In addition to surgical knowledge and practical skills, these should also teach competencies in differential diagnostics and treatment. The teaching of surgical knowledge through lectures and seminars and the demonstration of practical skills, e.g., through the use of logbooks in the Bock Practical Surgery (BP), only allows limited active engagement with surgical competencies on differential diagnostics and treatment. A reflection-based portfolio allows, through the independent written elaboration of surgical topics, an active engagement with the competencies and promises a higher learning effect. In the context of the implementation of such a portfolio as part of the proof of activity in BP, the effects on the acquisition of competencies and on the way of learning were investigated.

**Material and methods:**

Using a mixed methods approach, we compared competence acquisition using a reflection-based portfolio with learning using a logbook. Students conducted a self-assessment of competencies using questionnaire surveys before and after the BP. Through focus group interviews with discussions among students using a guideline, we explored the different ways of acquiring competencies. In addition, the examination and evaluation results of both cohorts were compared.

**Results and discussion:**

Students’ self-assessed competency acquisition and examination and evaluation results showed no differences when comparing the two cohorts. During the focus group interviews, we were able to show that in the perception of the students, surgical competencies can be made more visible and thus more explicit with the help of a reflection-based portfolio. In addition, self-regulated learning was promoted without neglecting practical skills. Students demanded greater supervision and guidance by mentors in both groups.

## Hintergrund

In Deutschland kommt es seit der Veröffentlichung des Nationalen Kompetenzbasierten Lernzielkataloges Chirurgie (NKLC) 2013, des Nationalen Kompetenzbasierten Lernzielkataloges Medizin (NKLM) 2015 und der neuen Weiterbildungsordnung 2020 zu einer zunehmenden Diskussion um eine kompetenzbasierte Ausbildung [13, 18]. Diese adressiert die Entwicklung von reiner Wissensvermittlung zur Vermittlung von Anwendungswissen, Fertigkeiten und Haltung, sie soll selbstreguliertes Lernen fördern und auf die Praxis vorbereiten [14, 28]. Entsprechende Anpassungen von Kurrikula werden weltweit gefordert [15] und deutsche Kurrikula wurden auf die Vermittlung praktischer Fertigkeiten der Chirurgie angepasst [25]. International wird die Vermittlung weiterer *chirurgischer Kompetenzen* wie Diagnosestrategien, Problemlösungen, das Verständnis von Indikationen für Interventionen, Nutzen, Grenzen, Risiken und mögliche Komplikationen von Verfahren und Therapien gefordert [1]. Es gibt Hinweise, dass dies in Deutschland noch in der initialen Entwicklungsphase steckt [24]. Die Herausforderung mag in der Unterrichtsmethodik liegen und neue Methoden müssen erprobt werden, um zukünftige Chirurg:innen auf das Fach vorzubereiten [4].

Das Blockpraktikum (BP) ist, laut der derzeitig gültigen Approbationsordnung von 2002, eine „Veranstaltung zur Differenzialdiagnostik und -therapie der wichtigsten Krankheitsbilder unter Bedingungen des klinischen und ambulanten Alltags“ [3]. Auch wenn das BP sehr unterschiedlich ausgestaltet werden kann, lernen Studierende, neben Seminaren, in der Regel den Alltag in der Chirurgie kennen [5]. Die Strukturierung der Lerninhalte bei der Vermittlung praktischer Fertigkeiten erfolgt oft über den quantitativen Nachweis in sog. Logbüchern [17]. Diese können den Lernfortschritt überwachen, selbstgesteuertes Lernen fördern und Lernen damit positiv beeinflussen [19].

Rückmeldungen auch aus der eigenen Fakultät legten den Verdacht nahe, dass das BP Chirurgie nicht in der Lage war, die notwendigen *chirurgischen Kompetenzen*, insbesondere im Hinblick auf die Differenzialdiagnostik, -therapie und chirurgischen Versorgungspfaden von Krankheitsbildern, zu vermitteln [29]. Die Implementation eines Portfolios im BP Chirurgie sollte diese *Kompetenzen* expliziter vermitteln und damit die Lücke schließen. Es ermöglicht z. B. durch eine zusätzliche schriftliche Erarbeitung beobachteter oder selbst durchgeführter klinischer Routinetätigkeiten im chirurgischen Alltag eine Reflexion des Gelernten [27]. Das Selbstlernen wird durch Beantwortung von Lernhilfefragen themenbezogen gesteuert und mithilfe von Feedback durch Mentor:innen gefördert [9]. Es ermöglicht die für einen hohen Lerneffekt notwendige aktive Auseinandersetzung mit Lernzielen, die Sammlung bearbeiteter Aufgaben bezüglich der geforderten *chirurgischen Kompetenzen* und das im klinischen Alltag üblicherweise eher indirekt Gelernte wird sichtbarer und greifbarer [7, 11, 16, 27]. Es wird empfohlen, die Effekte solcher Portfolios zu untersuchen und gleichzeitig sicherzustellen, dass die Änderung der Unterrichtsmethodik keine negativen Auswirkungen nach sich ziehen [6].

## Studiendesign und Untersuchungsmethoden

An unserer Fakultät war im Rahmen des BP Chirurgie bis zum Wintersemester 2018/2019 (WiSe 18/19) die Bearbeitung eines Logbuchs mit quantitativem Nachweis praktischer Fertigkeiten Pflicht (Tab. 1; [17]). Zusätzlich fanden Seminare und chirurgische Fallvorstellungen zur Differenzialdiagnostik und -therapie durch die Studierenden statt. Als objektive Prüfungsmethode erfolgte eine fächerübergreifende klinisch-praktische Prüfung, eine sog. OSCE(Objective Structural Clinical Examination)-Prüfung.

**Table 1:** 

Thema	Erforderliche Anzahl	Leistungsnachweis
Blutabnahme	Mind. 5 Blutabnahmen	□ Erbracht □ Nicht erbracht
Legen peripherer Verweilkanülen	Fakultativ	… gelegt
Anamnese und körperliche Untersuchung	Bei mind. 4 Patienten	□ Erbracht □ Nicht erbracht
Aufklärungsgespräch	Teilnahme an 4 Gesprächen + 1 schriftlicher Bericht	□ Erbracht □ Nicht erbracht
Operationsassistenz	Teilnahme an 4 bis 8 Operationen + 1 selbst verfasster Operationsbericht	□ Erbracht □ Nicht erbracht
Verbandswechsel	Mind. 4 Verbandswechsel	□ Erbracht □ Nicht erbracht
Arztbrief	Mind. 2 Arztbriefe verfasst	□ Erbracht □ Nicht erbracht

Prospektiv erfolgte im WiSe 2018/2019 eine anonyme Fragebogenerhebung vor dem PB Chirurgie (T1) und eine vergleichende Fragebogenerhebung (T2) sowie ein Studierendeninterview im Rahmen einer Fokusgruppe nach dem BP Chirurgie. Fokusgruppen sind eine qualitative Methode zur Erforschung und Erklärung von Themenkomplexen in der Medizinausbildung, in deren Rahmen Erfahrungen zu Auswirkungen von Lernformen erfasst werden können [26].

Unter Zuhilfenahme von Lernhilfefragen zu klinischen Routinetätigkeiten fordert das neu eingeführte Portfolio kleinschrittig zur schriftlichen Reflexion praktischer Fertigkeiten und *chirurgischer Kompetenzen* zu Differenzialdiagnostik und -therapie und Patient:innen-Management auf (Tab. 2; [6]).

**Table 2:** 

Themen des Portfolios zu klinischen Fertigkeiten	Themen zur der Differenzialdiagnostik und -therapie
Anamnese und klinische Untersuchung	Anamnese/Komorbiditäten
Blutabnahme/Legen von Venenverweilkanülen	Begründung der Diagnosestellung
Chirurgische Aufklärung	Begründung der Indikationsstellung
Operative Versorgung/inkl. Verfassen eines Operationsberichtes	Begründung der Operationsmethode
Wundversorgung und Hygiene	Mögliche Risiken und Komplikationen
Arztbriefschreibung	Begründung notwendiger prä- und postoperativer Maßnahmen
***Beispiele der Lernhilfefragen zur Reflexion:***	
Welche Punkte einer Anamnese helfen, einen schnellen Überblick über eine Erkrankung zu erhalten und den momentanen Allgemeinzustand zu erfassen?	Welche Befunde bestätigen die Diagnose?
Welche Inhalte sollte eine Aufklärung umfassen? Was versteht man dabei unter allgemeinen und speziellen Risiken?	Existieren Klassifikationen, Leitlinien, Richtlinien?
Nach welchen Kriterien wird eine Wunde beurteilt und wofür ist das relevant?	Begründen Sie die Indikationsstellung zum gewählten therapeutischen Verfahren!
Was ist eine Kausalitätskette/wozu ist sie wichtig?	
	Nennen Sie Risiken und mögliche Komplikationen (auch spezifische) des angewendeten operativen Verfahrens!
	Welche prä-/postoperativen Schritte zur Vorbereitung der Operation/Intervention sind notwendig? Nennen Sie Gründe!

Das Portfolio wurde als Pilot im Sommersemester 2019 (SoSe 19) und im Wintersemester 2019/20 (WiSe 19/20) fest implementiert. Aufgrund einer bekannten demografischen Heterogenität zwischen den Sommer- und Wintersemesterkohorten an unserer Fakultät erfolgten die nächsten Fragebogenerhebungen (T1 und 2) im WiSe 19/20 und Fokusgruppen im SoSe 19 und im WiSe 19/20. Die Ergebnisse der abschließenden OSCE-Prüfung und die fakultätseigene anonyme Online-Evaluation des BP Chirurgie über EVALuna in beiden WiSe wurden zusätzlich betrachtet.

Für die bessere Lesbarkeit werden die erhobenen Daten in den untersuchten Semestern im Ergebnisteil der jeweiligen Unterrichtsmethode zugeordnet. Damit wird das WiS 18/19 „Logbuch“ benannt und das SoSe 19 und WiSe 19/20 entsprechend „Portfolio“.

### Fragebogen

Die erste Fragebogenerhebung erfolgte in Form einer Selbsteinschätzung vor dem BP Chirurgie (T1) [12]. Es wurde, neben demografischen Daten, nach den Erwartungen an das BP Chirurgie für die Entwicklung der eigenen Kompetenzen als Ärzt:innen (Likert-Skala 1–6; 1 = sehr gut, 6 = ungenügend) und nach der Einschätzung, ob sie sich auf die Tätigkeit als Ärztinnen vorbereitet fühlen (Likert-Skala 1–6; 1 = stimme voll und ganz zu, 6 = stimme gar nicht zu) gefragt. Die Studierenden wurden zudem gebeten, ihr Wissen und ihre Kompetenzen zu praktischen Fertigkeiten und *chirurgischen Kompetenzen* (Likert-Skala 1–6; 1 = sehr gut, 6 = ungenügend) einzuschätzen und diese bezüglich der konkreten Lernziele des Logbuchs einzuordnen (Likert-Skala 1–4; 1 = ich beherrsche es nicht, 2 = ich kann es unter Aufsicht durchführen, 3 = ich kann es eigenständig durchführen, 4 = ich könnte es anderen vermitteln). Nach dem BP Chirurgie erfolgte jeweils eine vergleichende Fragebogenerhebung (T2).

## Statistik

Die Analyse der Daten erfolgte mittels STATA-Software (Version 17.0, DPC Software GmbH, Solingen). Die Daten zu den einzelnen Items wurden zunächst auf Normalverteilung geprüft und entsprechend mit einem parametrischen oder nichtparametrischen T‑test zweier unabhängiger Stichproben ausgewertet. Dargestellt werden die Mittelwerte, Standardabweichungen und die Signifikanz mit einem festgelegten Niveau von *p* < 0,05. Zusätzlich erfolgte die Berechnung des Deltas der Veränderungen der Mittelwerte der Items und die Berechnung des *P*(T ≤ t) zweiseitig und der t‑Statistik für die Darstellung eines möglichen Unterschiedes zwischen den Semestern.

### OSCE-Prüfung/Evaluation

Der Vergleich der OSCE-Ergebnisse des WiSe 18/19 (Logbuch) und des WiSe 19/20 (Portfolio) werden in Prozent dargestellt. Bei den drei OSCE-Stationen der Chirurgie handelte es sich um vergleichbare klinische Fertigkeiten: „körperliche Untersuchung“, „Wundnaht und korrektes hygienisches Verhalten“, „mündliche Aufklärung über einen kleinen operativen Eingriff“.

Die übliche Evaluation zur Zufriedenheit über „EVALuna“ (Onlineevaluationsinstrument der Binary Design GmbH, Münster) mit verschiedenen Aspekten des Unterrichts erfolgte in beiden Semestern (Likert-Skala 1–7; 1 = absolut unzufrieden bis 7 = absolut zufrieden); diese werden unter Angabe des Mittelwertes und des Medians dargestellt.

### Fokusgruppeninterviews

Die Interviews erfolgte im WiSe 18/19 (Logbuch) randomisiert mit 4 Studierenden (2 männlich, 2 weiblich), im Pilotsemester SoSe 19 (Portfolio) in 2 Interviews mit 8 (5 männlich, 3 weiblich) und im WiSe 19/20 (Portfolio) in 2 Interviews mit 7 (4 männlich und 3 weiblich) Teilnehmer:innen. Es sollten Effekte auf die Art und Weise des Lernens und der Weg des Kompetenzerwerbs durch ein reflexionsbasiertes Portfolio im Vergleich zu einem Logbuch untersucht werden. Anhand eines zuvor erstellten Leitfadens unter Beachtung von Erkenntnissen aus der Literatur, wurden Aussagen zu Erfahrungen zu den in Tab. 3 dargestellten Kategorien und Unterkategorien eingeholt. Die Auswertung erfolgte nach Erreichen der Sättigung von Themen durch die Teilnehmer durch eine qualitative Inhaltsanalyse [21]. Die Aussagen werden interpretativ dargestellt und in Auszügen beispielhaft zitiert.

**Table 3:** 

Kategorie	Unterkategorie	Kategorie	Unterkategorie
Unterrichtsform Blockpraktikum	Erwartungen an das BP	Lernziele allgemein	Bewusstsein
	Elemente des Unterrichts		Selbstreguliertes Lernen
			Einfluss Dozierende
			Aktive Einforderung
Vorwissen	Bewusstsein	Lernziele speziell	Lernerfolg
	Nutzen		Lernform
			Gelingen
			Herausforderungen

Frage-Kategorien der Fokusgruppen-Interviews

*BP* Blockpraktikum

## Ergebnisse

### Fragebogen

Im WiSe 18/19 (Logbuch) beantworteten zum Zeitpunkt T1 *n* = 90 und zum Zeitpunkt T2 *n* = 74 Studierende, im WiSe 19/20 (Portfolio) zum Zeitpunkt T1 *n* = 117 und zum Zeitpunkt T2 *n* = 90 Studierende den Fragebogen (Tab. 4). Insgesamt nahmen mehr weibliche als männliche Studierende an den Fragebogenerhebungen teil, was der Verteilung in der Gesamtkohorte entsprach. Im Durchschnittsalter waren die Semester ähnlich (im Schnitt 25 Jahre alt). Zum Zeitpunkt T1 zeigte sich bezüglich der Vorerfahrung in der Chirurgie kein wesentlicher Unterschied zwischen den Semestern. Zum Zeitpunkt T2 beantworteten im WiSe 19/20 (Portfolio) mehr Studierende mit Vorerfahrung (59 %) den Fragebogen als im WiSe 18/19 (Logbuch; 49 %). Diese ergaben sich hauptsächlich aus in der Chirurgie abgeleisteten Famulaturen.

**Table 4:** 

	Zeitpunkt	Anzahl	Alter (Jahre)	Geschlecht		Vorerfahrung in der Chirurgie	
				m (%)	w (%)	Ja (%)	Nein (%)
Logbuch	T1	90	25,7	31	69	51	49
	T2	74	25,7	35	65	56	54
Portfolio	T1	117	24,8	39	61	49	51
	T2	90	25,2	43	57	59	41

Vor (T1) und nach (T2) dem Blockpraktikum

*m* männlich, *w* weiblich

Die „Erwartungen an das BP“ (T1) und der „Wert des BP“ (T2) für die „Entwicklung der eigenen Kompetenzen“ wurde von beiden Semestern in etwa gleich bewertet und beide Semester zeigten nach dem BP eine signifikante Steigerung der Einschätzung bezüglich der „Vorbereitung auf die Tätigkeit als Ärzt:innen“ (Tab. 5). Die Studierenden beider Semester zeigten nach dem BP eine signifikante Verbesserung der Selbsteinschätzung bezüglich des Wissens und Kompetenzen ausgewählter klinisch-praktischer Fertigkeiten zu den Themen „Rund um die Operation“, „Wundversorgung/Hygiene im Operationssaal“, „Tätigkeiten auf Station, inkl. Hygiene“ und „Arztbriefschreibung“ (Tab. 5). Allein die Kompetenz bezüglich der „Patientenaufklärung unter Supervision“ zeigte sich im WiSe 18/19 (Logbuch) signifikant und im WiSe 19/20 (Portfolio) nicht signifikant gebessert.

**Table 5:** 

Item	Semester	Zeitpunkt	MW ± SD	*p*
Vorbereitung auf die Tätigkeit als Ärzt:innen (1)	Logbuch	T1	3,92 ± 1,17	0,0000^a^
		T2	3,04 ± 1,31	
	Portfolio	T1	3,52 ± 1,14	0,0000^a^
		T2	2,49 ± 1,14	
Erwartungen und Wert des BP für die Entwicklung der eigenen Kompetenzen als zukünftige Ärtz:innen (2)	Logbuch	T1	2,27 ± 0,87	0,0066^b^
		T2	2,77 ± 1,26	
	Portfolio	T1	2,38 ± 0,85	0,0025^b^
		T2	2,78 ± 1,12	
Wissen um die Indikation zur operativen Therapie (2)	Logbuch	T1	3,49 ± 0,99	0,0001^b^
		T2	2,92 ± 0,84	
	Portfolio	T1	3,25 ± 0,88	0,0063^a^
		T2	2,89 ± 0,99	
Wissen um die Durchführung von Operationen (2)	Logbuch	T1	4,14 ± 1,17	0,0001^a^
		T2	3,45 ± 1,1	
	Portfolio	T1	3,8 ± 1,21	0,0411^a^
		T2	3,46 ± 1,21	
Wissen um die prä- und postoperative Versorgung von Patient:innen (2)	Logbuch	T1	3,46 ± 1,08	0,0015^b^
		T2	2,91 ± 0,1	
	Portfolio	T1	3,31 ± 1,05	0,0002^a^
		T2	2,77 ± 0,95	
Wissen um Risiken und Komplikationen chirurgischer Eingriffe (2)	Logbuch	T1	3,17 ± 0,97	0,0001^b^
		T2	2,58 ± 0,83	
	Portfolio	T1	2,88 ± 0,88	0,0175^a^
		T2	2,58 ± 0,92	
Kompetenz bez. Patient:innen-Aufklärung (unter Supervision) vor operativen Eingriffen (2)	Logbuch	T1	3,06 ± 0,94	0,0010^b^
		T2	2,64 ± 0,96	
	Portfolio	T1	2,97 ± 1,04	0,1186^a^
		T2	2,73 ± 1,08	
Kompetenz bez. Verfassen eines Operationsberichtes (2)	Logbuch	T1	4,31 ± 1,19	0,0000^b^
		T2	3,51 ± 1,25	
	Portfolio	T1	4,13 ± 1,12	0,0000^a^
		T2	3,27 ± 1,04	
Kompetenz bez. Wundversorgung (2)	Logbuch	T1	3,33 ± 1,14	0,0000^b^
		T2	2,5 ± 0,95	
	Portfolio	T1	3,13 ± 1,08	0,0016^a^
		T2	2,64 ± 1,07	
Wissen um Hygienestandards (2)	Logbuch	T1	2,4 ± 0,95	0,0000^b^
		T2	1,88 ± 0,84	
	Portfolio	T1	2,46 ± 1,09	0,0040^b^
		T2	2,08 ± 1,0	
Erkennen und Benennen unsterilen Verhaltens im Operationssaal (2)	Logbuch	T1	2,28 ± 1,04	0,0000^b^
		T2	1,53 ± 0,81	
	Portfolio	T1	2,27 ± 1,1	0,0000^b^
		T2	1,71 ± 0,89	
Wissen um die Bestandteile eines Arztbriefes (2)	Logbuch	T1	3,26 ± 0,95	0,0000^b^
		T2	2,24 ± 0,82	
	Portfolio	T1	3,17 ± 1,13	0,0000^a^
		T2	2 ± 0,78	
Kompetenz bez. Verfassen eines Arztbriefes (2)	Logbuch	T1	3,62 ± 0,95	0,0000^b^
		T2	2,62 ± 0,82	
	Portfolio	T1	3,35 ± 1,18	0,0000^a^
		T2	2,32 ± 0,76	

Ergebnisse des Fragebogens vor *(T1)* und nach *(T2)* dem Blockpraktikum, auf einer *(1)* Likert-Skala 1–6; 1 = stimme voll und ganz zu, 6 = stimme gar nicht zu, *(2)* Likert-Skala 1–6; 1 = sehr gut, 6 = ungenügend; Zweistichproben T‑Test: *MW* *+* *SD* Mittelwert ± Standardabweichung

*p* = Signifikanzniveau < 0,05

^a^Normalverteilt

^b^Nicht normalverteilt

In der Selbsteinschätzung zu konkreten praktischen Fertigkeiten des Logbuchs zeigte auch das WiSe 19/20 (Portfolio) unter der Anleitung durch ein reflexionbasiertes Portfolio, ohne quantitative Nachweise, in allen Fertigkeiten eine signifikante Verbesserung (Tab. 6).

**Table 6:** 

Item	Semester	Zeitpunkt	MW ± SD	*p*
Blutabnahme	Logbuch	T1	3,49 ± 0,62	0,0001^b^
		T2	3,81 ± 0,46	
	Portfolio	T1	3,50 ± 0,65	0,0000^b^
		T2	3,8 ± 0,62	
Legen von Venenverweilkanülen	Logbuch	T1	3,1 ± 0,8	0,0148^b^
		T2	3,39 ± 0,74	
	Portfolio	T1	3 ± 0,94	0,0026^b^
		T2	3,36 ± 0,87	
Verbandswechsel	Logbuch	T1	2,31 ± 0,88	0,0000^a^
		T2	3,09 ± 0,67	
	Portfolio	T1	2,51 ± 0,83	0,0470^a^
		T2	2,76 ± 0,92	
Chirurgische Anamnese	Logbuch	T1	2,4 ± 0,64	0,0000^a^
		T2	2,99 ± 0,61	
	Portfolio	T1	2,45 ± 0,7	0,0011^b^
		T2	2,81 ± 0,82	
Chirurgische Untersuchung	Logbuch	T1	1,9 ± 0,60	0,0000^b^
		T2	2,66 ± 0,76	
	Portfolio	T1	2,07 ± 0,67	0,0000^b^
		T2	2,59 ± 0,82	
Steriles Einwaschen	Logbuch	T1	2,63 ± 1,02	0,0000^b^
		T2	3,66 ± 0,53	
	Portfolio	T1	2,69 ± 0,1	0,0000^b^
		T2	3,56 ± 0,75	
Wundnaht	Logbuch	T1	1,91 ± 0,88	0,0000^b^
		T2	2,61 ± 0,9	
	Portfolio	T1	2,04 ± 0,99	0,0007^a^
		T2	2,49 ± 0,85	

Ergebnisse des Fragebogens vor *(T1)* und nach *(T2)* dem Blockpraktikum auf einer Likert-Skala 1–4 (1 = ich beherrsche es nicht, 2 = ich kann es unter Aufsicht durchführen, 3 = ich kann es eigenständig durchführen, 4 = ich könnte es anderen vermitteln). Zweistichproben T‑Test: *MW* *+* *SD* Mittelwert ± Standardabweichung

*p* = Signifikanzniveau < 0,05

^a^Normalverteilt

^b^Nicht normalverteilt

Das Lernen mit Logbuch mit Schwerpunkt auf dem quantitativen Nachweis wie auch das Lernen mit Portfolio führten zu einer Verbesserung der Selbsteinschätzung bezüglich praktischer Fertigkeiten und *chirurgischer Kompetenzen*. Im statistischen Vergleich der beiden Methoden konnte kein signifikanter Unterschied im Delta der Veränderung des Mittelwertes zwischen den Semestern festgestellt werden (Tab. 7).

**Table 7:** 

	Semester	∆ MW ± SD	*p*	t‑Statistik	Kritischer t‑Wert
Veränderung der Selbsteinschätzung zu „Wissen und Kompetenzen zu klinisch-praktischen Fertigkeiten“	Logbuch	0,70 ± 0,04	0,2630	1,146	2,063
	Portfolio	0,58 ± 0,10			
Veränderung der Selbsteinschätzung zu „praktischen Fertigkeiten des Logbuchs“	Logbuch	0,64 ± 0,07	0,1549	1,518	2,179
	Portfolio	0,44 ± 0,04			

Nach Bestimmung des Delta *(∆)* der Mittelwertveränderungen in beiden Gruppen; T‑Test: ∆ *MW* *+* *SD* Mittelwert ± Standardabweichung

*p* = Signifikanzniveau < 0,05 (kritischer t‑Wert > t-Statistik = keine Signifikanz)

### Fokusgruppeninterviews

Mithilfe der Fokusgruppen wurden Erfahrungen zur Art und Weise des Kompetenzerwerbs bezüglich der im Folgenden vorangestellten Themen als Schwerpunkte ermittelt.

#### Erwartungen

– Studierende beider Semester erhofften sich das Kennenlernen des Arbeitsalltags in der Chirurgie, das Erlernen praktischer Fertigkeiten und das Vertiefen und Anwenden von Wissen. Die Erwartungen wurden grundsätzlich erfüllt. Die Unterstützung durch Mentor:innen war eher gering.

#### Logbuch

- *„… meine Erwartung war eigentlich, dass wir die Theorie, die wir vorher gelernt haben, … jetzt praktisch umsetzen.“,*
- „*im klinischen Alltag, sieht man, was wirklich wichtig ist …“*
- *„… dass die (Stationsärzte) nicht die Zeit hatten …“*

#### Portfolio

- *„… Vertiefen und Anwenden von Wissen …!“*
- *„… vor allem Neues lernen …“*

#### Vorwissen

 – Studierenden mit Logbuch war dies nicht bewusst. Die Studierenden mit Portfolio haben es durch die aktive Auseinandersetzung bewusst eingefordert und sich auch außerhalb des Unterrichts mit den Themen auseinandergesetzt. Positiv wurden die resultierende Wiederholung und das Abprüfen des eigenen Vorwissens bewertet. Die Reflexion habe zu einem aktiven Austausch untereinander geführt.

#### Logbuch

- *„… nicht bekannt, höchstens aus Vorlesungen …“*

#### Portfolio

- „*Das benötigte Vorwissen war klar …“*
- *„… es hat auch im klinischen Alltag genützt!“*

#### Lernziele des BP

 – Studierenden mit Logbuch waren diese (Tab. 1) teilweise bekannt, sie wurden aber nicht bewusst verfolgt. Es fehlte zudem Feedback. Studierende mit Portfolio fühlten sich gut angeleitet und angehalten, zu üben. Sie erreichten die Lernziele ebenfalls durch viel Eigeninitiative. Sie wurden im Peer-Verfahren und interprofessionell erarbeitet. Positiv wurde die inhaltliche Auseinandersetzung durch das Portfolio bewertet, bemängelt wurde die fehlende Supervision.

#### Logbuch

- *„… ich habe nicht eine Anamnese gemacht! … nicht unter ärztlicher Aufsicht!“*
- *„… musste mich selber drum bemühen …“*

#### Portfolio

- *„Man wurde nicht persönlich angehalten, aber durch die Aufgaben im Portfolio wurde man dazu angehalten!“*
- *„Sehr ausführliche Anleitung und man musste sich halt selbst darum kümmern!“*
- *„… wenig Supervision oder Korrekturen …!“*

#### Indikationsstellung und prä- und postoperative Vorbereitung

 – Studierende mit Logbuch lernten dies nicht bewusst. Sie beschrieben eine Wahrnehmung im Alltag. Den Studierenden mit Portfolio waren diese Lernziele explizit bekannt. Sie nutzten das Portfolio, um sich mit der Thematik zu beschäftigen, für die Vor- und Nachbereitung und für Feedback durch Dozierende.

#### Logbuch

- *„… das haben wir im Alltag so mitbekommen …!“*
- *„… nie aktiv mitbekommen …“*

#### Portfolio

- *„Doz. haben Portfolio herangezogen …haben Feedback gegeben!“*
- *„durch das Portfolio … dadurch haben wir uns damit beschäftigt …“*
- *„… durch das Portfolio ist es klar geworden, es wurde verinnerlicht …!“*

#### Schreiben eines Operationsberichtes

 – Wurde durch das Logbuch wenig angeleitet und mit Portfolio, trotz Anleitung, als schwierig empfunden.

#### Logbuch

- *„..ich habe Textbausteine benutzt …“*
- *„… andere haben einfach abgeschrieben!“*

#### Portfolio

- „*Portfolio hat gut für das Schreiben … angeleitet… insgesamt aber schwierig!“*

#### Verbandswechsel und Wundbeurteilung; Hygienestandards

 – Wurden mit Logbuch oft durchgeführt und „mit wenig Teaching“ kommentiert. Mit Portfolio wurde es teilweise oft, teilweise gar nicht durchgeführt. Das Portfolio habe geholfen, sich mit dem Thema und seiner Relevanz auseinanderzusetzen.

#### Logbuch

- *„… habe ich ganz viel gemacht, aber es gab wenig Teaching …“*

#### Portfolio

- *„Portfolio hat geholfen sich nochmal Schritt für Schritt damit auseinanderzusetzen …und die Wichtigkeit nochmals gefestigt …!“*

#### Arztbriefschreibung

 – Studierende mit Logbuch kommentierten wenig Wissenszuwachs. Studierende mit Portfolio nannten die Anleitung und die geforderte Auseinandersetzung eine gute Hilfestellung und betonten gleichzeitig die geringe Betreuung durch die Mentor:innen.

#### Logbuch

- *„Mir ist immer noch nicht ganz klar was da rein muss …“*
- *„… wenn man es machen muss, bekommt man Übung!“*

#### Portfolio

- *„… Eher wenig oder keine Hilfe von Assistenzärzten… durch die Vorgaben im Portfolio konnte die Aufgabe trotzdem bewältigt werden“*

#### Verbesserungsvorschläge

 – Studierende mit Logbuch wünschten sich eine aktivere Auseinandersetzung mit den Lernzielen und mehr Kontakt zu den Mentor:innen. Studierende mit Portfolio wünschten mehr theoretischen Unterricht, mehr klinische Fälle, konkrete Besprechungen von Operationen und Operationsberichten und ein Arztbriefseminar. Auch sie wünschten mehr Supervision.

#### Logbuch

- *„… Lernziele sollten so als Syllabus in das Logbuch …, kann man sich dann dran lang hangeln, oder aktiv einfordern!“*
- *„… mehr Kontakt zu Assistenzärzten …“*

#### Portfolio

- *„… mehr theoretische Unterricht … mehr Patientenfälle …“*
- *„… mehr Supervision… mehr Beachtung.“*
- *„Arztbriefseminare wären gut …“*

Insgesamt zeigten Studierende mit Portfolio ein explizites Bewusstsein der Lernziele und bestätigten eine aktive Auseinandersetzung mit den Inhalten. Es kam zu einer Reflexion und Interaktion mithilfe der positiv bewerteten Anleitung. Den Studierenden mit Logbuch dagegen waren die Lernziele teilweise nicht bekannt, sie bemängelte die fehlende Anleitung und wünschten sich eine bewusstere Auseinandersetzung mit den Themen. Beide Gruppen wünschten sich eine stärkere Supervision und Feedback.

### OSCE/Evaluation

Die Ergebnisse der objektiven OSCE-Prüfung und die fakultätseigene Evaluation zeigten keinen relevanten Unterschied zwischen dem Semester mit Logbuch und dem Semester mit Portfolio (Tab. 8 und 9). Das Semester mit Logbuch zeigte sich mit der Prüfungsvorbereitung gering besser zufrieden als das Semester mit Portfolio, welches dagegen bezüglich der Hilfsmittel für die Lehrveranstaltung eine etwas höhere Zufriedenheit aufwies (Tab. 8).

**Table 8:** 

	Logbuch (%)	Portfolio (%)
*Gesamt*	*83*	*82*
Station C1	79	77
Station C2	93	90
Station C3	75	79

*OSCE* Objective Structured Clincal Examination; Bestehensgrenze: 60 %

**Table 9:** 

Evaluation der Zufriedenheit mit	Logbuch		Portfolio	
	Median	MW	Median	MW
**Lehrveranstaltung insgesamt**	**5**	**4,3**	**5**	**4,4**
Inhalte der Lehrveranstaltung	5	4,9	5	4,6
Organisation der Lehrveranstaltung	5	4,7	5	4,5
*Prüfungsvorbereitung der Lehrveranstaltung*	**5**	4,5	**4**	4,2
Leistung der Dozierenden	5	5,0	5	4,7
Rahmenbedingungen (Räume, techn. Ausstattung etc.)	5	5,2	5	4,9
*Hilfsmitteln der Lehrveranstaltung (z.* *B. Skript, Folien etc.)*	**4**	4,2	**5**	4,5
Subjektiver Wissenszuwachs	5	4,7	5	4,3

Likert-Skala 1–7; 1 = absolut unzufrieden bis 7 = absolut zufrieden

*MW* Mittelwert, *EvaLuna* Onlineevaluationsinstrument der Binary Design GmbH Münster

## Diskussion

Die Empfehlung des Wissenschaftsratens (2018) zu einer stärkeren Kompetenzorientierung im Medizinstudium für die Umsetzung des „Masterplans 2020“ führt zu einem Paradigmenwechsel von der fachbasierten Vermittlung von Wissensinhalten zu einer stärkeren Kompetenzorientierung in Lehre und Prüfungen [30]. Der NKLC der Deutschen Gesellschaft Chirurgie versucht, die chirurgischen Themen diesbezüglich noch sichtbarer und anwendbar zu machen [18] und auch die neue Weiterbildungsordnung rückt die Kompetenzorientierung in den Fokus. Medizinstudierende sollten ein Verständnis für chirurgische Versorgungspfade und für die Optimierung der Patientenversorgung entwickeln, mit Schwerpunktlegung auf die wichtigsten Verfahren [20]. Dies gelingt am ehesten in klinischer Lernumgebung, in der die Standardisierung von Lernzielen allerdings schwierig ist [1].

Die Gewährleistung der Umsetzung praktischer Fertigkeiten erfolgt oft über den quantitativen Nachweis in Logbüchern [17]. Die dabei eher geringe inhaltliche Auseinandersetzung kann zu einem „Abarbeiten“ der Tätigkeiten mit einem eher geringen Lerneffekt führen [2]. Unsere Untersuchung zeigt, dass das Lernen mit einem Logbuch einerseits zu einer signifikanten Verbesserung der Selbsteinschätzung der Studierenden bezüglich chirurgischer Fertigkeiten und Kompetenzen geführt hat, aber gleichzeitig Defizite in der inhaltlichen Auseinandersetzung mit den Lernzielen bestehen [2]. Die Lernziele wurden nicht bewusst erreicht und eine tiefere Auseinandersetzung damit wurde gefordert.

Die für einen hohen Lerneffekt notwendige aktive Auseinandersetzung kann ein begleitendes Portfolio bewirken [7, 11, 16]. Der Erwerb von Wissen in kleinen Teilstücken, eine Reflexion und die Möglichkeit von Interaktion ist dabei wesentlich [7, 9]. Dies ist im Klinikalltag eine Herausforderung [10, 22]. Bei ähnlicher Zusammensetzung der untersuchten Semester, konnten wir zeigen, dass die Ergebnisse der Prüfung und die Evaluation vergleichbar waren und dass das Lernen mit einem Portfolio zu einer ebenso signifikanten Verbesserung der Selbsteinschätzung bezüglich chirurgischer Fertigkeiten und Kompetenzen geführt hat wie das Lernen mit Logbuch.

Portfoliobasiertes Lernen soll spezifische Lernziele verfolgen, die sich an den Kurszielen ausrichten, auf die zukünftige ärztliche Tätigkeit vorbereiten und mit klaren Aufgabenstellungen zu bearbeiten sein [6]. Es ist dabei in der Lage, theoretisches Wissen mit praktischem Wissen in Verbindung zu bringen, es führt zur Selbsterkenntnis und ermöglicht zusätzlich eine Bewertung des Gelernten [7]. Weiterhin ermöglicht es in der Regel ein hohes Engagement von Studierenden und wird in der Medizinausbildung überwiegend positiv bewertet [8]. Dies ist auch auf andere Standorte und unabhängig vom Lehrkonzept anwendbar [7].

Auch unsere Studierenden sahen in dem Portfolio eine Hilfestellung für die aktive Auseinandersetzung und Einordnung der Inhalte. Dies hat, in der Wahrnehmung der Studierenden, zu einem vertieften Lernen geführt, die Interaktion im klinischen Alltag gefördert und Inhalte wurden im Vergleich zum logbuchgesteuerten Lernen explizit gelernt [6]. Mangelnde personelle und zeitliche Ressourcen in der Betreuung von Studierenden sind bekannte Herausforderungen beim Unterricht im klinischen Alltag [10]. Eine bessere Supervision und Feedback wurden von den Studierenden unabhängig von der Methode gefordert. Positive Effekte von Portfolios können dadurch verstärken werden [9].

Eine Limitation dieser Untersuchung stellt die fehlende Validierung des Fragebogens für die Erfassung *chirurgischer Kompetenzen* zu Differenzialdiagnostik und -therapie dar. Die vergleichende Selbsteinschätzung ist nicht unumstritten, kann aber den longitudinalen Nachweis von Kompetenzerwerb unterstützen [12]. Sie spiegelt objektiv gemessene Leistungsniveaus wider und reagiert auf Lehränderungen [23]. In der Kombination mit den Fokusgruppen [12] konnte beim Lernen mit Portfolio in der Chirurgie die stärkere Auseinandersetzung mit den *chirurgischen Kompetenzen* aufgezeigt werden, was mit Blick auf die Literatur einen höherer Lerneffekt erwarten lässt [2, 16].

## Fazit für die Praxis

- Ein Lernen mit Portfolio im Blockpraktikum Chirurgie
  - führt zu einer vergleichbaren Verbesserung der Selbsteinschätzung bezüglich *chirurgischer Kompetenzen* und praktischer Fertigkeiten wie ein Lernen mit Logbuch,
  - fördert selbstreguliertes studentisches Lernen,
  - ermöglicht eine explizitere und tiefere Auseinandersetzung mit *chirurgischen Kompetenzen*.
- Supervision und Feedback sollte (unabhängig von der Methode) ermöglicht werden.
